# Transcranial Magnetic Stimulation‐Induced Modulation of Functional Connectivity in Healthy Controls: A TMS–EEG Graph Study

**DOI:** 10.1002/brb3.70981

**Published:** 2025-12-22

**Authors:** Inés Fernández‐Linsenbarth, Gema Mijancos‐Martínez, Saúl J. Ruiz‐Gómez, Alejandro Bachiller, Emma Osorio‐Iriarte, Rosa M. Beño‐Ruiz‐de‐la‐Sierra, Antonio Arjona‐Valladares, Alejandro Roig‐Herrero, Vicente Molina

**Affiliations:** ^1^ Psychiatry Department School of Medicine University of Valladolid Valladolid Spain; ^2^ Biomedical Engineering Research Centre (CREB) Department of Automatic Control (ESAII) Polytechnic University of Catalonia Barcelonatech (UPC) Barcelona Spain; ^3^ Institute of Research Sant Joan de Déu Barcelona Spain; ^4^ Imaging Processing Laboratory University of Valladolid Valladolid Spain; ^5^ Psychiatry Service Clinical Hospital of Valladolid Valladolid Spain; ^6^ Neurosciences Institute of Castilla y León (INCYL) University of Salamanca Salamanca Spain

**Keywords:** functional connectivity, graph theory, small‐world, TMS–EEG

## Abstract

**Introduction:**

The combination of transcranial magnetic stimulation (TMS) and electroencephalography (EEG) enables direct interaction with the brain while recording the resulting neural activity, offering a unique opportunity to understand the brain's functional dynamics. In this study, TMS–EEG was employed to examine the effects of TMS on functional network connectivity through graph theory parameters.

**Methods:**

A total of 29 healthy controls underAuthor: Please check funding information and confirm its correctness.went a single‐pulse TMS–EEG protocol targeting the dorsolateral prefrontal cortex. Three graph theory parameters summarizing functional network properties (i.e., connectivity strength, clustering coefficient, and characteristic path length) were analyzed before and after TMS application.

**Results:**

TMS‐single pulse administration over the dorsolateral prefrontal cortex of healthy controls was associated with a significant increase in connectivity strength and clustering coefficient, and a significant decrease in the characteristic path length parameters. These changes are consistent with a shift towards a small‐world network organization.

**Conclusion:**

These findings provide insight into the neurophysiological mechanisms underlying TMS‐induced changes and could have potential therapeutic implications.

## Graphical abstract

TMS was associated with a modulation of functional network connectivity. TMS modulation effects seem to reflect a change into a small‐world organization. EEG‐based brain graphs contribute to understand TMS neurophysiological basis.

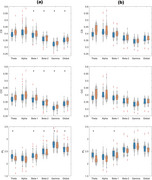

## 1 Introduction

Simultaneous transcranial magnetic stimulation (TMS) and electroencephalography (EEG) have become valuable methods offering essential understanding of brain function and dynamics (Farzan et al. 2016). The delivery of a TMS pulse to the cortex induces a time‐locked depolarization of underlying neurons, along with trans‐synaptic activation of both nearby and distant cortical networks, which can be assessed with EEG (Tremblay et al. 2019). Multiple TMS–EEG protocols have been developed to evaluate neural excitation, inhibition, plasticity, and oscillatory activity across different brain regions in both healthy and diseased states (Cao et al. 2021; Farzan et al. 2016; Hui et al. 2019). However, to our knowledge, no previous study has explored the effect of TMS on functional network connectivity through graph theory parameters in healthy subjects. Graph theory views the brain as an intricate, interconnected system (E. Bullmore and Sporns 2009; Friston 2011; Sporns 2018) composed of nodes linked by edges (E. T. Bullmore and Bassett 2011), providing a unique approach for examining functional network connectivity. In brain graphs derived from EEG, electrodes serve as nodes, whereas edges (i.e., synchrony) are estimated through coupling metrics (Stam and van Straaten 2012). According to graph theory, it has been suggested that brain networks exhibit a small‐world (SW) architecture (Sporns 2011; Yu et al. 2008), defined by a balanced integration and segregation (i.e., communication between long‐distance and local regions, respectively) which would facilitate parallel information transmission (Finotelli and Dulio 2015; Strogaz 2001). Therefore, assessing the effect of TMS on functional connectivity using EEG‐based graph theory parameters may be particularly advantageous, as it could provide additional evidence of the excitability changes produced throughout the cortex in response to magnetic stimulation.

Previous evidence suggests that magnetic stimulation influences functional network connectivity in healthy individuals, but it primarily stems from studies using repetitive TMS (rTMS). Thus, rTMS has been associated with an alteration of the functional connectivity strength between different cortical regions in healthy controls (HCs) based on coherence metrics (Jing and Takigawa 2000; Plewnia et al. 2008) and functional magnetic resonance imaging (Gromann et al. 2012). Moreover, rhythmic TMS promotes local synchronization of natural brain oscillations, emulating the oscillatory activity generated by cognitive tasks (Thut et al. 2011). Additionally, it has been shown that functional network connectivity increases from theta to gamma bands in the resting state networks of HCs when using transcranial direct current stimulation (tDCS) applied to the dorsolateral prefrontal cortex (DLPFC) (Polanía et al. 2011). Moreover, significant changes in functional connectivity through network analysis methods have also been shown in HCs combining rTMS and motor training (Jin et al. 2017). Regarding clinical populations, rTMS has been associated with a significant increase in weighted node degree (i.e., average functional connectivity between sensors by means of synchrony) in patients with depression (Cosmo et al. 2023), as well as postherpetic neuralgia patients (Pei et al. 2019). Based on these findings, the study of how TMS affects the network properties of functional connectivity in healthy individuals using graph theory parameters could aid in the identification of the neuronal processes underlying TMS. It is important to note here that rTMS consists of delivering sequences of magnetic pulses at different frequencies, which, when administered with suitable timing, length, and strength, are expected to induce changes in synaptic efficacy that persist beyond the stimulation period (Tremblay et al. 2019). Because of this, rTMS is increasingly being used as a complementary therapy to promote plasticity. However, single TMS pulses have been more widely used for the study of brain function (Klomjai et al. 2015).

The primary aim of this study was to explore the impact of TMS on functional connectivity networks using graph theory parameters (i.e., connectivity strength [CS], clustering coefficient [ClC], and characteristic path length [PL]). For this purpose, we analyzed the characteristics of the graph theory parameters before and after applying TMS‐single pulses over the DLPFC in theta, alpha, beta‐1, beta‐2, gamma, and global frequency bands. Subsequently, we analyzed baseline and response to TMS graph theory parameters and characteristics. The rationale behind studying functional connectivity modulation with TMS over the DLPFC stems from evidence indicating that this region is crucial for several higher‐order processes (Miller and Cohen 2001), and alterations in DLPFC function are often observed in neuropsychiatric disorders such as schizophrenia, addiction, and depression (Feil et al. 2010; Gonzalez‐Burgos et al. 2011; Goto et al. 2010; Lewis et al. 2011). We hypothesize that TMS over the DLPFC will transiently modulate global functional brain connectivity.

## 2 Methods

### 2.1 Participants

A total of 29 right‐handed HC (mean age = 26.5 ± 11.4 years, range = 18–54 years; 13 males and 16 females) participated in the study. This sample partially overlaps with the one used in two earlier published studies from our research group (Fernández‐Linsenbarth et al. 2024; Mijancos‐Martínez et al. 2024). Exclusion criteria included: (a) an intelligence quotient under 70; (b) a history of drug or alcohol abuse; (c) a self‐reported comorbid medical illness; (d) receiving any treatment affecting the central nervous system; and (f) not being safe to undergo TMS. Handedness was confirmed using the Oldfield Handedness Inventory (Oldfield 1971). All participants gave their written informed consent after full written information and the study was endorsed by the local ethics committee of the Clinical University Hospital of Valladolid, in accordance with the ethical standards in the Helsinki Declaration of 1975, as revised in 2008.

### 2.2 Transcranial Magnetic Stimulation

TMS was performed using a MagPro ×100 stimulator (MagVenture, Denmark) and a figure‐of‐8 coil (MCF‐B70). During the experiment, participants were seated comfortably and asked to look directly ahead with their eyes open. At the start of the study, an EEG cap was positioned on their head, and electrodes were attached over the right abductor pollicis brevis (APB) muscle for electromyographic recording. Subsequently, the resting motor threshold (RMT) was determined over the left motor cortex using the relative frequency method (Groppa et al. 2012), which is defined as the lowest intensity needed to produce a motor evoked potential (MEP) of > 50 µV peak‐to‐peak amplitude in at least 5 out of 10 subsequent trials. Thereafter, 75 single pulses of monophasic TMS were administered at an intensity of 120% RMT over the left DLPFC, utilizing a semi‐randomized inter‐stimulus interval of 5–7 s, to avoid predictability of the upcoming pulse. During stimulation, the coil was placed at the midpoint between the F3 and F5 electrodes, allowing for a precise estimation of the left DLPFC and minimal intersubject variability in the absence of neuronavigational tools (Fitzgerald et al. 2009; Rogasch et al. 2015; Rusjan et al. 2010). The handle of the coil was oriented backward at approximately 45° to the midsagittal line, inducing a posterior–anterior current flow in the brain tissue beneath. To assess auditory‐evoked potentials that might interfere with the cortical TMS response of interest, 23 participants underwent sham stimulation protocols. Sham stimulation was applied using the same protocol as active stimulation but by rotating the TMS coil by 90° in relation to the left DLPFC, following previous studies (Du et al. 2017; Rogasch et al. 2014).

### 2.3 EEG Recording

EEG signals were acquired using a 64‐channel amplifier system (Brain Vision [Brain Products GmbH]) in accordance with the 10–10 international system. An ActiChamp amplifier model alongside active electrodes compatible with TMS was used. Two of the channels were positioned on the outer side of each eye to track eye movement artifacts. All electrodes were referenced to the Cz electrode during data collection, and the impedance for every electrode was reduced to ≤ 5 kΩ. EEG signals were recorded at a sampling rate of 25 kHz, which has been demonstrated to reduce the TMS‐related artifact (Daskalakis et al. 2008).

### 2.4 TMS–EEG Data Processing

TMS–EEG data were processed using MATLAB (R2021b; The Mathworks Inc., Natick, MA) and Fieldtrip (Oostenveld et al. 2011). The procedure was analogous to the one performed in (Mijancos‐Martínez et al. 2024). Signals were epoched around the TMS pulse, including 1000 ms of prestimulus baseline and 1000 ms of poststimulus activity. Data samples from −1 to 10 ms related to the TMS pulse were eliminated and cubic interpolated (Rogasch et al. 2014) because of their irretrievable nature. The data was subsequently re‐referenced to common average. Artifact reduction was performed by implementing an independent component analysis (ICA), after which three experts manually identified the independent components associated with muscle, ocular, auditory, TMS‐induced, and noise‐related artifacts based on trial‐averaged amplitude, time‐frequency maps, and spatial distribution and activation maps (Cline et al. 2021; Rogasch et al. 2015, 2014). This was followed by an automatic bad channel interpolation and bad trial rejection. Finally, the baseline was corrected using an interval of 800 ms preceding the TMS pulse onset, resampled to 5 kHz, and a band‐pass filter ranging from 0.5 Hz to 70 Hz was applied. Sham stimulation signals were processed analogously to the active stimulation signals.

### 2.5 Graph Theory Parameters

Graph theory parameters were calculated for both active and sham stimulation signals via the phase locking value (PLV) across successive trials using the continuous wavelet transform (CWT) method. PLV is calculated by measuring the variability in phase difference over successive trials (Lachaux et al. 1999), making this measure sensitive to low amplitude oscillations (Spencer et al. 2003) and nonlinear behaviors (van Diessen et al. 2015). In mathematical terms, it is defined as (Lachaux et al. 1999):

$$\mathrm{PL}V_{xy}\;\left(k,s\right)=\frac{1}{Nt}\;\left|\sum_{n=1}^{N}e^{\Delta \varphi_{xy}\left(k,s,n\right)}\right|,$$

where *Nt* is the number of trials, $\Delta \varphi_{xy}$ represents the instantaneous phase difference between the signals *x* and *y*, *k* indicates the time interval, and *s* is the scaling factor of the mother wavelet. In this study, both *k* and *s* were set to 1, in order to obtain a balanced relationship between temporal and frequency resolution at low frequencies.

**FIGURE 1:**
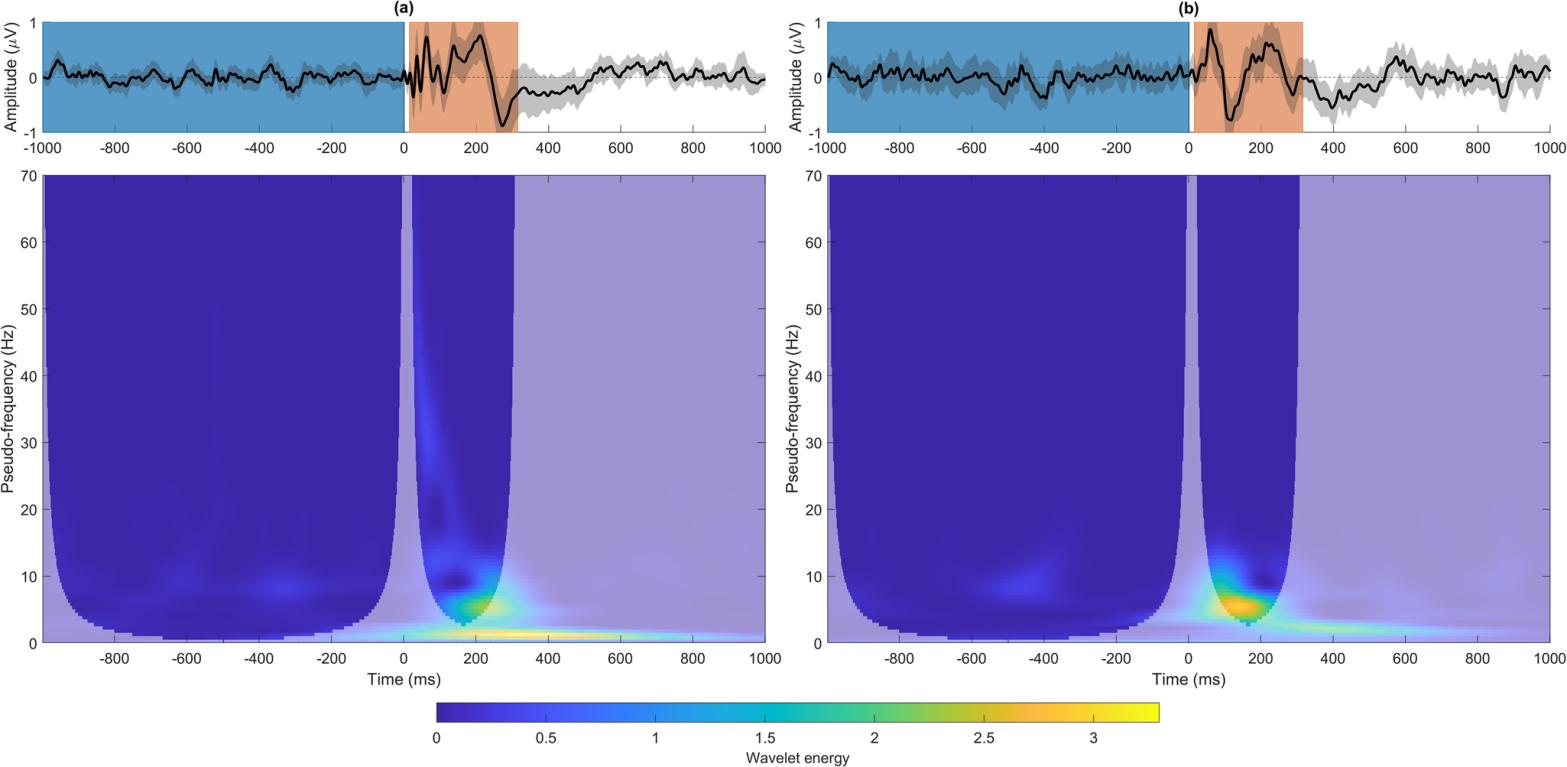
Grand‐averaged raw ERPs and wavelet scalogram across trials and subjects at the DLPFC electrodes for (a) active stimulation and (b) sham stimulation. In the ERP representations, the solid black lines indicate the average values, while the shaded areas represent the standard deviation across all subjects. Prestimulus and poststimulus windows are indicated in blue and orange, respectively. The scalograms were computed by squaring the absolute value of the CWT coefficients across trials and subjects. The cone of interest (COI) for prestimulus and poststimulus windows, where spectral content is not affected by edge effects, is represented by a transparency mask overlaid over the scalogram.

The segmentation of EEG signals results in our recordings being finite and short‐time, leading to a variation in the wavelet energy due to the discontinuity at signal edges (Torrence and Compo 1998). To determine the Heisenberg boxes that remain unaffected by edge effects, a cone of influence (COI) can be defined (Torrence and Compo 1998). In this study, two different COIs were assessed as trials were decomposed into two temporal windows: (i) the prestimulus window, which corresponded to a baseline period before the TMS pulse from −1000 ms to the TMS pulse; and (ii) the poststimulus window from 15 to 315 ms following the TMS pulse. The choice of the poststimulus window is justified following previous literature showing this time window coinciding with when the EEG responses phase‐locked to the TMS, known as TMS‐evoked potentials (TEPs), occur (Farzan et al. 2016; Hill et al. 2016; Tremblay et al. 2019). Figure 1 illustrates the grand‐averaged raw evoked response potentials (ERPs) and wavelet scalograms displaying the prestimulus and poststimulus COIs across trials and subjects at the DLPFC electrodes for both active and sham stimulation.

PLV was computed in the standard EEG frequency bands: theta (θ, 4–8 Hz), alpha (α, 8–13 Hz), beta‐1 (β1, 13–19 Hz), beta‐2 (β2, 19–30 Hz), and gamma (γ, 30–70 Hz), as well as in the global band (4–70 Hz). Delta band (δ, 1–4 Hz) was excluded due to the absence of fully enclosed Heisenberg boxes within the response COI (a window length longer than 300 ms is required).

Using the PLV connectivity matrices, the three network parameters (i.e., CS, ClC, and PL) that summarize various dimensions of both global and local brain connectivity (Rubinov and Sporns 2010) were calculated in the prestimulus and the response windows.

CS summarizes the average edge values for all nodes within the network, and it was calculated as follows:

$$\mathrm{CS}=\frac{\sum_{i=1}^{N}\sum_{j\;>i}w_{ij}}{N\left(N\;-\;1\right)/2},$$

where $w_{ij}$ represents the PLV values between nodes *i* and *j*, and *N* denotes the total number of nodes (Gomez‐Pilar et al. 2018).

ClC is a metric for segregation based on the number of triangles in the network, which measures the brain's capability to process information within densely interconnected hubs. It is calculated as the proportion of node neighbors that are also mutual neighbors (Rubinov and Sporns 2010):

$$\mathrm{ClC}=\frac{1}{N}\sum_{i\in N}\frac{2t_{i}}{s_{i}\left(s_{i}-1\right)},$$

where $t_{i}$ represents the geometric mean of triangles surrounding node *i* and $s_{i}$ denotes the strength of node *i*.

PL is a measure of functional integration that quantifies the ability to merge specialized information from various brain areas. It is defined as the mean shortest path length between all node pairs within the network (Rubinov and Sporns 2010):

$$\mathrm{PL}=\frac{1}{N}\Sigma_{i\in N}\frac{\sum_{j\in N}l_{ij}}{N\;-\;1},$$

where $l_{ij}$ represents the shortest path length in distance between nodes *i* and *j*.

Finally, to control for the effect of volume conduction and confounds introduced by common‐average referencing, we decided to check whether the effects found in the active stimulation were also present using the corrected imaginary PLV (ciPLV) measure, which corrects for these confounding factors:

$$\mathrm{ciPL}V_{xy}\;\left(t\right)=\frac{\frac{1}{T}ℑ\left\{e^{-i\left(\varphi_{x}\left(t\right)-\varphi_{y}\left(t\right)\right)}\right\}}{\sqrt{1-{\left(\frac{1}{T}ℜ\left\{e^{-i\left(\varphi_{x}\left(t\right)-\varphi_{y}\left(t\right)\right)}\right\}\right)}^{2}}},$$

where *T* is the data length, ℑ represents the imaginary part, ℜ represents the real part, and φ is the instantaneous phase of the signals *X* and *Y* at time *t*. As can be inferred from the formula, ciPLV remove the contribution of the zero phase differences of PLV that could be affected by volume conduction effects, as almost “zero‐lag” interactions are considered as noise affecting real zero‐lag interactions.

### 2.6 Statistical Analyses

First, a paired‐samples *t*‐test was performed to examine the change of EEG graph theory parameters (i.e., CS, ClC, and PL) across each frequency band between the prestimulus and the poststimulus temporal windows. The Bonferroni correction was applied to reduce the risk of Type 1 errors (three graph theory parameters and six frequency bands; *p *= 0.002). Second, to rule out the potential influence of auditory evoked potentials caused by magnetic stimulation on the results, a paired‐sample *t*‐test was conducted with sham stimulation data for those relationships that were identified as significantly different in the first analysis. Similarly, the Bonferroni correction was applied to minimize the risk of Type 1 errors (three graph theory parameters, five frequency bands; *p* = 0.003). All statistical analyses were conducted using SPSS 29.0.1 (SPSS Inc., Chicago, IL, USA).

## 3 Results

### 3.1 TMS Effect on Graph Theory Parameters

**FIGURE 2:**
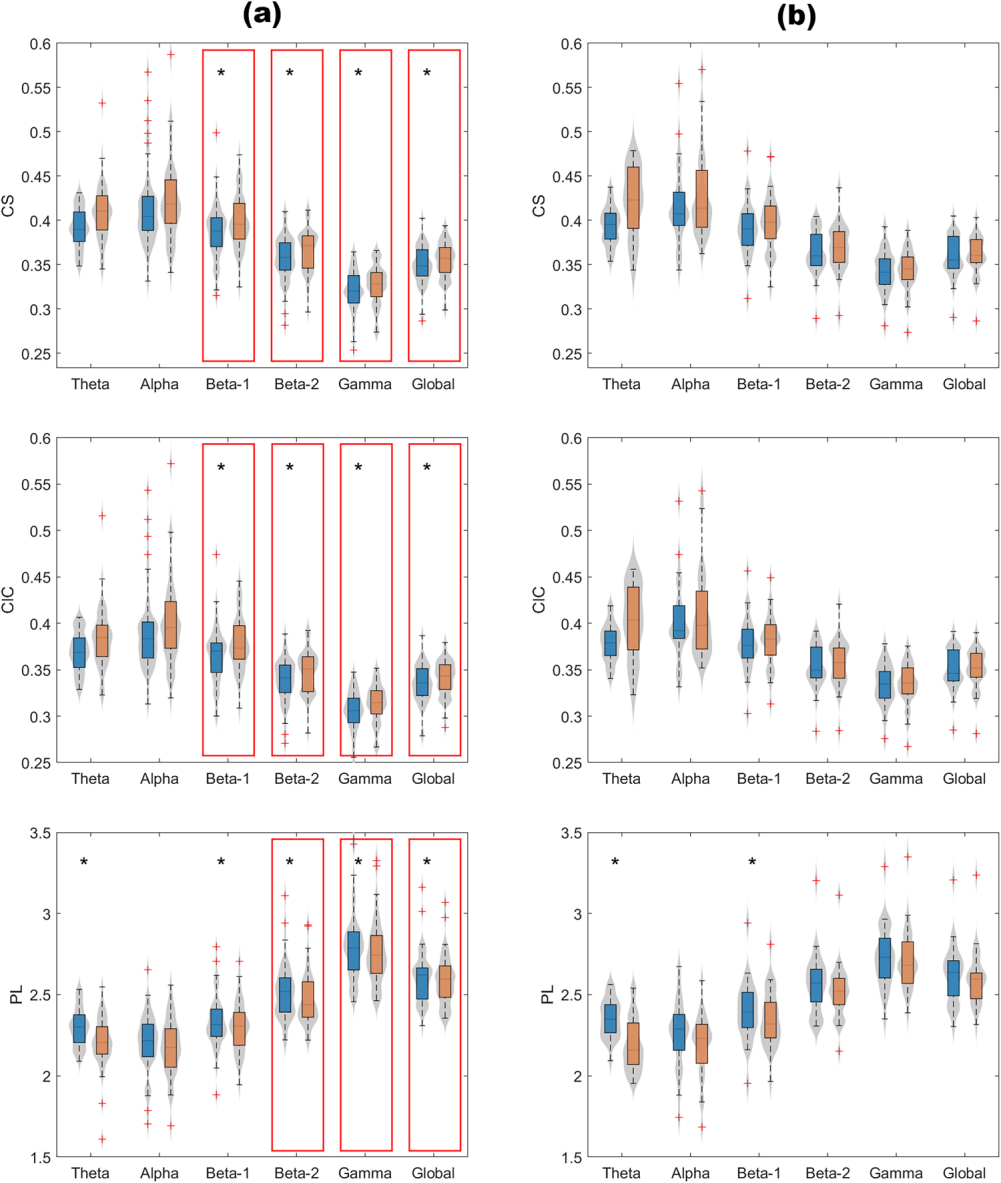
Boxplots displaying the CS, ClC, and PL values distributions at each frequency band in the prestimulus (blue) and poststimulus (orange) windows for (a) active stimulation and (b) sham stimulation. Statistical analyses were performed using a paired‐sample *t*‐test. Only statistical differences that survive the Bonferroni correction are marked (*p < 0.002 for active stimulation and *p* < 0.003 for sham stimulation). The red rectangles highlight significant differences that are exclusively for active stimulation but not for sham stimulation.

Participants showed a statistically significant increase in CS and ClC along with a significant decrease in the PL parameter across all frequency bands after undergoing TMS‐pulses (Table 1, Figure 2a). Following the Bonferroni correction, the increase in CS and ClC remained significant for the fast bands (i.e., beta‐1, beta‐2, and gamma) as well as the broadband. The decrease in the PL parameter remained significant across all frequency bands, excluding alpha.

**TABLE 1:** Graph theory parameters characteristics assessed with PLV before and after TMS‐pulses administration.

	Prestimulus	Poststimulus	Statistic	*p* value	Cohen's *d*
Connectivity strength					
Theta	0.39 (0.02)	0.40 (0.03)	*t*(28) = −2.76	0.005	0.02
Alpha	0.41 (0.05)	0.42 (0.05)	*t*(28) = −2.24	0.017	0.03
Beta‐1	0.38 (0.04)	0.40 (0.03)	*t*(28) = −4.49	< 0.001^a^	0.02
Beta‐2	0.35 (0.03)	0.36 (0.03)	*t*(28) = −4.17	< 0.001^a^	0.01
Gamma	0.32 (0.02)	0.33 (0.02)	*t*(28) = −5.53	< 0.001^a^	0.01
Global	0.35 (0.02)	0.35 (0.02)	*t*(28) = −3.64	< 0.001^a^	0.01
Clustering coefficient					
Theta	0.37 (0.02)	0.38 (0.03)	*t*(28) = −2.01	0.027	0.02
Alpha	0.39 (0.05)	0.40 (0.05)	*t*(28) = −2.06	0.025	0.03
Beta‐1	0.36 (0.03)	0.38 (0.03)	*t*(28) = −4.35	< 0.001^a^	0.02
Beta‐2	0.34 (0.02)	0.35 (0.02)	*t*(28) = −4.11	< 0.001^a^	0.01
Gamma	0.30 (0.02)	0.31 (0.02)	*t*(28) = −5.17	< 0.001^a^	0.01
Global	0.33 (0.02)	0.34 (0.02)	*t*(28) = −3.49	< 0.001^a^	0.01
Characteristic path length					
Theta	2.31 (0.11)	2.25 (0.13)	*t*(28) = 3.65	< 0.001^a^	0.09
Alpha	2.23 (0.20)	2.19 (0.20)	*t*(28) = 2.62	0.007	0.09
Beta‐1	2.36 (0.18)	2.29 (0.16)	*t*(28) = 4.47	< 0.001^a^	0.08
Beta‐2	2.53 (0.18)	2.48 (0.18)	*t*(28) = 3.84	< 0.001^a^	0.07
Gamma	2.82 (0.21)	2.76 (0.19)	*t*(28) = 5.55	< 0.001^a^	0.05
Global	2.63 (0.17)	2.59 (0.17)	*t*(28) = 3.87	< 0.001^a^	0.04

*Note*: Data are given as mean (standard deviation).

^a^ Statistically significant after Bonferroni correction (i.e., *p* < 0.002).

Regarding the ciPLV measure, the analysis showed analogous results to the PLV measure. After the Bonferroni correction, the increase in CS and ClC remained significant in the same frequency bands except gamma, and the decrease in PL remained significant in all frequency bands except gamma (Table 2).

**TABLE 2:** Graph theory parameters characteristics assessed with ciPLV before and after TMS‐pulses administration.

	Prestimulus	Poststimulus	Statistic	*p* value	Cohen's *d*
Connectivity strength					
Theta	0.08 (0.01)	0.10 (0.02)	*t*(28) = −4.13	< 0.001^a^	0.02
Alpha	0.09 (0.02)	0.11 (0.04)	*t*(28) = −3.38	0.001	0.03
Beta‐1	0.08 (0.01)	0.10 (0.02)	*t*(28) = −4.91	< 0.001^a^	0.02
Beta‐2	0.08 (0.01)	0.09 (0.01)	*t*(28) = −4.69	< 0.001^a^	0.01
Gamma	0.08 (0.01)	0.08 (0.01)	*t*(28) = −2.93	0.003	0.01
Global	0.08 (0.01)	0.09 (0.01)	*t*(28) = −5.63	< 0.001^a^	0.01
Clustering coefficient					
Theta	0.09 (0.01)	0.10 (0.02)	*t*(28) = −2.42	0.011	0.02
Alpha	0.10 (0.02)	0.11 (0.03)	*t*(28) = −2.84	0.004	0.03
Beta‐1	0.09 (0.01)	0.10 (0.02)	*t*(28) = −4.29	< 0.001^a^	0.02
Beta‐2	0.09 (0.01)	0.09 (0.01)	*t*(28) = −4.23	< 0.001^a^	0.01
Gamma	0.08 (0.01)	0.09 (0.01)	*t*(28) = −2.64	0.007	0.01
Global	0.09 (0.01)	0.09 (0.01)	*t*(28) = −5.42	< 0.001^a^	0.01
Characteristic path length					
Theta	12.33 (1.55)	9.38 (1.59)	*t*(28) = 8.26	< 0.001^a^	1.92
Alpha	11.45 (2.29)	8.91 (2.29)	*t*(28) = 5.21	< 0.001^a^	2.62
Beta‐1	12.34 (1.44)	9.84 (1.61)	*t*(28) = 6.12	< 0.001^a^	2.20
Beta‐2	12.68 (1.44)	11.51 (1.49)	*t*(28) = 4.99	< 0.001^a^	1.27
Gamma	12.74 (1.06)	12.46 (1.21)	*t*(28) = 1.83	0.039	0.81
Global	12.42 (0.90)	11.71 (1.00)	*t*(28) = 5.06	< 0.001^a^	0.75

*Note*: Data are given as mean (standard deviation).

^a^ Statistically significant after Bonferroni correction (i.e., *p* < 0.002).

### 3.2 Sham Stimulation Effect on Statistically Significant Relationships

Sham stimulation was not associated with statistically significant changes between baseline and response conditions in graph theory parameters, except for the theta and beta‐1 bands of the PL parameter (see Table 3, Figure 2b).

**TABLE 3:** Graph theory parameters characteristics assessed with PLV before and after TMS sham stimulation.

	Prestimulus	Poststimulus	Statistic	*p* value
Connectivity Strength				
Beta‐1	0.38 (0.03)	0.39 (0.03)	*t*(22) = −2.12	0.023
Beta‐2	0.36 (0.03)	0.37 (0.02)	*t*(22) = −1.29	0.105
Gamma	0.33 (0.02)	0.34 (0.02)	*t*(22) = −0.92	0.184
Global	0.36 (0.02)	0.36 (0.02)	*t*(22) = −1.14	0.134
Clustering coefficient				
Beta‐1	0.38 (0.03)	0.38 (0.03)	*t*(22) = −1.33	0.099
Beta‐2	0.35 (0.02)	0.35 (0.02)	*t*(22) = −0.82	0.212
Gamma	0.33 (0.02)	0.33 (0.02)	*t*(22) = −0.26	0.397
Global	0.35 (0.02)	0.35 (0.02)	*t*(22) = −0.78	0.222
Characteristic path length				
Theta	2.37 (0.11)	2.19 (0.17)	*t*(22) = 6.92	< 0.001^a^
Beta‐1	2.41 (0.19)	2.37 (0.18)	*t*(22) = 3.13	0.002^a^
Beta‐2	2.58 (0.19)	2.53 (0.17)	*t*(22) = 2.29	0.016
Gamma	2.75 (0.18)	2.72 (0.19)	*t*(22) = 2.19	0.020
Global	2.64 (0.18)	2.62 (0.18)	*t*(22) = 1.79	0.044

*Note*: Data are given as mean (standard deviation).

^a^ Statistically significant after Bonferroni correction (i.e., *p *< 0.003).

## 4 Discussion

Our study explored the modulatory effects of TMS on functional network connectivity in healthy participants. The administration of suprathreshold TMS‐single pulses over the DLPFC was associated with a modulation of functional network connectivity, as assessed using graph theory parameters. Specifically, TMS induced a significant increase in CS and ClC and a significant decrease in the characteristic path length parameters. In other words, the administration of TMS‐single pulses over the DLPFC was linked to enhanced connectivity density, segregation, and functional integration, particularly within the faster frequency bands.

The modulation of functional connectivity by TMS, as revealed through graph theory parameters reported here, seems to reflect a shift towards an SW organization, characterized by increased CS and ClC, and reduced PL. SW networks combine high local efficiency (clustering) with short average path lengths (global efficiency), which is thought to enhance information transmission processes (Strogaz 2001) by promoting synchronization across brain regions. These findings support the idea that brain functional networks seem to operate in a critical dynamic state, enabling a rapid reconfiguration of network topology in response to swiftly changing cognitive demands (Shafi et al. 2012). Furthermore, our results seem to align with the idea that functional networks in the brain are flexible, with different cognitive states being associated with changes in the weight of functional connections (Bortoletto et al. 2015).

The findings of this study may have important therapeutic implications. Previous evidence indicates that various psychiatric and neurological disorders are associated with abnormal neural synchronization (Uhlhaas and Singer 2006) and alterations in graph theory metrics (Cea‐Cañas et al. 2020; Gomez‐Pilar et al. 2017). Moreover, disruptions in the synchronization of oscillatory activity have been linked to cognitive impairments in schizophrenia and major depressive disorder (Chen et al. 2014; Fingelkurts and Fingelkurts 2015; Senkowski and Gallinat 2015). Consequently, precisely timed interventions that could modulate functional connectivity may induce neural changes that could promote some relief or improvement in these disorders. This is particularly relevant when considered in the context of our findings, in which the modulatory effects of TMS on functional network connectivity are especially remarkable in the faster bands, traditionally associated with the coordination of fundamental neural processes underlying cognition (Andrade et al. 2024; Fries 2009). In this regard, further research is needed to explore TMS‐induced modulation of functional connectivity as a potential target for therapeutic interventions, as well as to assess the long‐term effects of such modulation.

Compared to active stimulation, results showed that sham stimulation was not associated with significant changes in the graph parameters studied, except for the PL parameter in the theta and beta‐1 bands. While this is an unexpected outcome, previous research shows that placebo stimulation protocols in which the coil is rotated 90 degrees relative to the stimulation site yield neurophysiological responses significantly different from those resulting from active stimulation (Poorganji et al. 2021). Specifically, sham stimulation has been linked to lower amplitude waveforms in comparison to active stimulation (Bonato et al. 2006; Du et al. 2017; Rogasch et al. 2014), suggesting that sham stimulation could produce a certain degree of cortical stimulation, albeit significantly less than the active one, potentially due to residual peripheral magnetic fields from the coil's perpendicular alignment. Therefore, it seems coherent to expect that sham stimulation would induce changes in graph theory parameters, although of a much smaller magnitude than those produced by active stimulation, as our results indicate. Thus, while auditory evoked potentials contribute to the TMS response observed, they cannot entirely explain the TMS–EEG signal and the results obtained.

Certain limitations of our study should be noted. First, the sample size was relatively small. A bigger sample size would be preferable. Second, no neuronavigational system was employed to localize the left DLPFC. Nevertheless, the coil was placed in a spot that allows for a precise estimation of this brain area, following earlier studies in the field (Fitzgerald et al. 2009; Rogasch et al. 2015; Rusjan et al. 2010). This could have introduced some variability in coil positioning. However, we believe that this would have had a limited influence on the observed effects, given the robustness of the results at the global network level. Third, PLV analyses may not be free from volume conduction or common average confounds. Therefore, the results of this measure should be interpreted with caution. Moreover, the results observed in the theta band may be subject to spectral distortions due to the length of the temporal window analyzed. Therefore, the effects reported in this band should also be interpreted with caution. Fourth, our study comprised a single session during which participants were administered single pulses of TMS. To enhance the understanding of TMS‐induced modulation of functional connectivity through brain graph parameters, future studies could assess long‐lasting effects of TMS on functional network connectivity through TMS–EEG, which could have significant therapeutic implications. Finally, certain reported effect sizes were small, particularly in the CS and ClC metrics. This could be due to intersubject variability and/or the length of the time window used for the analysis, which may not have been long enough. A longer analysis time window would be desirable in future studies, which would in turn allow the exploration of interesting aspects such as the temporality of changes in network functional connectivity.

In conclusion, our study indicates that the application of single TMS pulses over the DLPFC of healthy individuals is associated with the modulation of functional network connectivity, offering novel insights into the physiological mechanisms underlying cortical changes induced by magnetic stimulation. Additionally, the findings of this study reinforce the value of TMS–EEG as a powerful neurophysiological tool for assessing several cortical properties, including functional connectivity through graph theory parameters.

## Author Contributions

**Inés Fernández‐Linsenbarth**: conceptualization, methodology, data curation, formal analysis, validation, investigation, writing – original draft. **Gema Mijancos‐Martínez**: formal analysis, methodology, software, writing – review and editing. **Saúl J. Ruiz‐Gómez**: formal analysis, methodology, software, writing – review and editing. **Alejandro Bachiller**: formal analysis, methodology, software, writing – review and editing. **Emma Osorio‐Iriarte**: data curation, investigation. **Rosa M. Beño‐Ruiz‐de‐la‐Sierra**: investigation, writing – review and editing. **Antonio Arjona‐Valladares**: investigation, writing – review and editing. **Alejandro Roig‐Herrero**: investigation. **Vicente Molina**: conceptualization, funding acquisition, project administration, resources, supervision, writing – review and editing.

## Ethics Statement

All participants gave their written informed consent after full written information, and the study was endorsed by the local ethics committee of the Clinical University Hospital of Valladolid, in accordance with the ethical standards in the Helsinki Declaration of 1975, as revised in 2008.

## Conflicts of Interest

The authors declare no conflicts of interest.

## Data Availability

The data that support the findings of this study are available from the corresponding author upon reasonable request.
